# Avian Influenza A(H5) Subtype in Wastewater — Oregon, September 15, 2021–July 11, 2024

**DOI:** 10.15585/mmwr.mm7406a5

**Published:** 2025-02-27

**Authors:** Rebecca Falender, Tyler S. Radniecki, Christine Kelly, Paul Cieslak, David Mickle, Harrison Hall, Ryan Scholz, Melissa Sutton

**Affiliations:** ^1^Oregon State University, Corvallis, Oregon; ^2^Oregon Health Authority; ^3^Oregon Department of Agriculture, Salem, Oregon.

**Summary**

**What is already known about this topic?**

Highly pathogenic avian influenza A(H5N1) outbreaks have emerged in U.S. cattle and poultry. Wastewater surveillance detects influenza A(H5) subtype but does not currently distinguish between human and animal sources.

**What is added by this report?**

During September 15, 2021–July 11, 2024, retrospective analysis of wastewater surveillance data revealed 21 avian influenza A(H5) subtype detections across 12 Oregon communities. No association was found between detections in a community’s wastewater and history of a poultry outbreak or presence of dairy processing facilities or dairy farms within the sewershed. Avian influenza A(H5) was detected most frequently in two communities with important wild bird habitats.

**What are the implications for public health practice?**

Wastewater surveillance was an early indicator of avian influenza emergence in Oregon. Nonhuman and noncattle animal inputs, including wild birds, are an essential consideration when interpreting A(H5) subtype detections in wastewater.

## Abstract

Wastewater surveillance is an important tool in the surveillance of emerging pathogens and has been leveraged during the highly pathogenic avian influenza (HPAI) A(H5N1) virus outbreak in cattle and poultry in the United States. Interpretation of avian influenza A(H5) subtype detections in wastewater requires an understanding of human and animal contributors to the sewershed because current testing does not distinguish between human and animal sources. Potential animal contributors include wild birds, farms with poultry or dairy cattle outbreaks, and dairy processing facilities. Retrospective analysis of 551 influenza A virus–positive wastewater surveillance samples from 20 sites in Oregon during September 15, 2021–July 11, 2024, revealed 21 avian influenza A(H5) subtype detections across 12 communities. Avian influenza A(H5) subtype detections in wastewater began approximately 6 weeks before Oregon’s first HPAI outbreak in domestic poultry, 7 weeks before Oregon’s first avian influenza A(H5) detection in wild birds, and 2 years before the first HPAI A(H5N1) outbreak in dairy cattle in the United States (Oregon has not detected HPAI A(H5N1) in dairy cattle or milk). No association was found between detection of avian influenza A(H5) in a community’s wastewater and history of an HPAI A(H5) outbreak among poultry in the county or presence of dairy processing facilities or dairy farms within the sewershed. Avian influenza A(H5) was detected most frequently in two communities with important wild bird habitats. Animal inputs, including from wild birds, should be considered when interpreting avian influenza A(H5) subtype detections in wastewater.

## Introduction

In January 2022, highly pathogenic avian influenza (HPAI) A(H5N1) clade 2.3.4.4b virus, originating in Europe, was first detected in wild birds in the United States (*1*). Since then, clade 2.3.4.4b has rapidly become enzootic in wild birds throughout North America and has demonstrated the ability to efficiently infect domestic poultry and some mammals (*1*). The first commercial poultry outbreak of HPAI A(H5N1) clade 2.3.4.4b in the United States occurred in February 2022 and, as of December 5, 2024, more than 112 million domestic birds in 49 states had been affected nationally (*2*). The first outbreak of HPAI A(H5N1) in U.S. dairy cattle occurred in March 2024 and, as of December 5, 2024, a total of 718 herds in 15 states had been affected nationally (*3*).

As part of the United States’ response to HPAI A(H5N1) outbreaks in animals and human cases and exposures, CDC is collaborating with state and local health departments to use wastewater surveillance to monitor influenza A virus and the avian influenza A(H5) subtype (*4*). Wastewater surveillance strengthens traditional case-based surveillance methods by providing community-level data independent of symptom status, health care–seeking behavior, and testing access, and can be quickly leveraged to detect emerging pathogens (*4*,*5*). However, interpretation of avian influenza A(H5) subtype detections in wastewater requires an understanding of human and animal contributors to the sewershed because current polymerase chain reaction (PCR)–based avian influenza A(H5) testing does not distinguish between human and animal sources (*4*). Potential animal contributors to wastewater include wild birds, farms with poultry or dairy cattle outbreaks, and dairy processing facilities (*4*,*6*).

In Oregon, clade 2.3.4.4b was first detected in wild birds and poultry in May 2022 and, as of December 5, 2024, a total of 44 HPAI outbreaks among poultry and none among dairy cattle have been identified (*2*,*3*). This report describes detections of the avian influenza A(H5) virus subtype in wastewater before and after HPAI was detected in wild birds and poultry in Oregon and dairy cattle in the United States.

## Methods

### Data Source and Study Design

Oregon tested wastewater seasonally for influenza viruses during September 15, 2021–June 24, 2022, and August 28, 2022–April 30, 2023, and has conducted year-round wastewater surveillance for influenza viruses since October 1, 2023. All influenza A virus–positive wastewater samples from a sample of 20 Oregon communities were retrospectively tested for the avian influenza A(H5) subtype. Communities were selected for geographic representativeness, migratory bird stopover activity, historic detections of avian influenza in wild birds, and the presence of licensed Grade A dairy processors* and farms within the sewershed.

### Specimen Collection and Laboratory Testing

As part of Oregon’s routine influenza A virus wastewater surveillance, 24-hour composite samples are collected from wastewater treatment facility influents 1–2 times weekly (*5*), filtered and then stabilized in DNA/RNA Shield (Zymo Research, https://www.zymoresearch.com). All wastewater samples are collected in duplicate, with the second filtered sample being archived at −112°F (−80°C). For this study, RNA was extracted from archived wastewater samples that previously tested positive for influenza A virus (*5*). Samples were analyzed for the avian influenza A(H5) subtype by digital reverse transcription PCR using the QIAcuity Eight instrument with the QIAcuity 26k 8-well Nanoplate (containing 26,000 partitions per sample) and the QIAcuity OneStep Advanced Probe Kit (QIAcuity, https://www.qiagen.com), per the manufacturer’s instructions. Primers (forward primer: TATAGARGGAGGATGGCAGG and reverse primer: ACDGCCTCAAAYTGAGTGTT) and probe (AGGGGAGTGGKTACGCTGCRGAC) were used (*6*). The one-step PCR cycling conditions were as follows: reverse transcription at 122°F (50°C) for 40 minutes; enzyme inactivation at 203°F (95°C) for 2 minutes; and 40 cycles that consisted of denaturation at 203°F (95°C) for 5 seconds followed by annealing and extension at 140°F (60°C) for 30 seconds. After PCR cycling, the 26,000 partitions were imaged on the HEX channel. Data from digital PCR imaging were analyzed using consistent thresholds, and quality control was ensured with positive (controlled at 100 or more positive partitions) and no-template^†^ (controlled at zero positive partitions) controls. All samples and quality controls were analyzed in duplicate.

### Statistical Analyses

The Fisher’s exact test was used to examine the association between avian influenza A(H5) subtype detections in wastewater and the presence of Grade A dairy processing facilities or dairy farms within the sewershed and reported poultry outbreaks within the county; p-values <0.05 were considered statistically significant. RStudio software was used to conduct all analyses (version 4.3.1; RStudio, Inc.). This activity was reviewed by Oregon Health Authority, deemed not research, and was conducted consistent with federal law and CDC policy.^§^

## Results

### Regional Characterization

**FIGURE 1:**
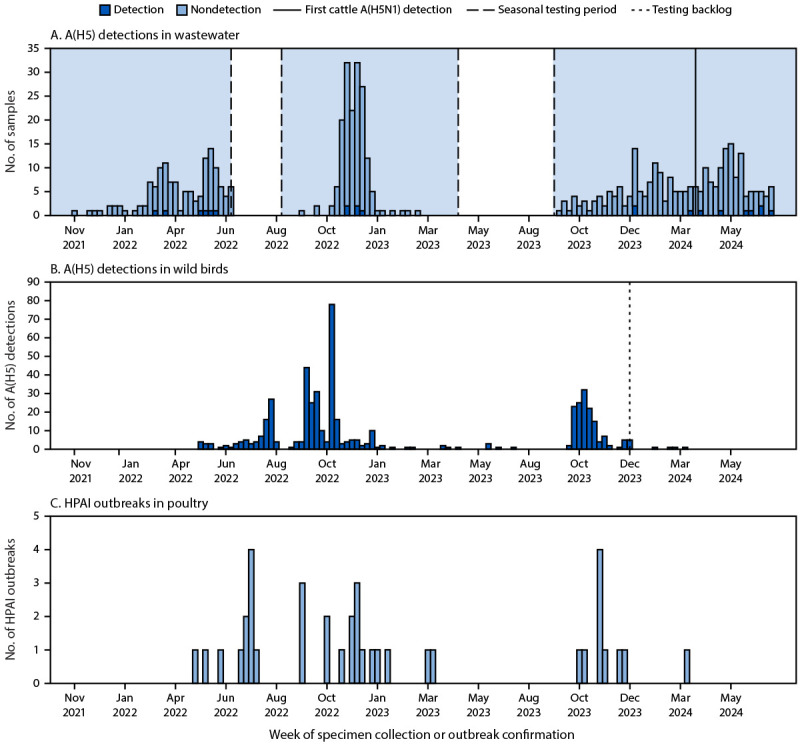
Number of detections of avian influenza A(H5) in wastewater (A)* and wild birds (B)^†^ and highly pathogenic avian influenza outbreaks in poultry (C)^§^ — Oregon, September 15, 2021–July 11, 2024^¶^ **Abbreviation:** HPAI = highly pathogenic avian influenza. * During June 2022–August 2023, influenza testing of wastewater was only conducted seasonally. ^†^ U.S. Department of Agriculture Animal and Plant Health Inspection Service has a backlog in testing wild bird samples, which might result in underrepresentation of the number of samples testing positive for avian influenza A(H5) after December 2023. https://www.aphis.usda.gov/livestock-poultry-disease/avian/avian-influenza/hpai-detections/wild-birds ^§^ https://www.aphis.usda.gov/livestock-poultry-disease/avian/avian-influenza/hpai-detections/commercial-backyard-flocks ^¶^ The first cattle HPAI A(H5N1) detection in the United States was not in Oregon. Oregon has not detected HPAI A(H5N1) in dairy cattle or milk.

**FIGURE 2:**
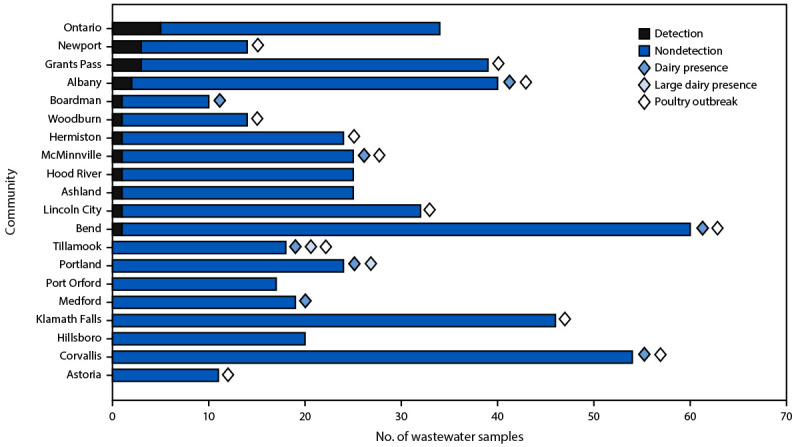
Number of wastewater samples tested for avian influenza A(H5), communities within a county that have experienced a highly pathogenic avian influenza A(H5) outbreak, and sewershed dairy presence* or large dairy presence^†^ — Oregon, September 15, 2021–July 11, 2024 * Community with a licensed Grade A dairy processor (a facility that produces milk under sufficiently sanitary conditions to qualify for human consumption) or dairy farm within the sewershed. ^†^ The sewersheds with the largest number of dairy farms (Tillamook [13]) and dairy processors (Portland [seven]).

Among 551 influenza A virus−positive samples during September 15, 2021–July 11, 2024, 21 (3.8%) tested positive for the avian influenza A(H5) subtype (Figure 1) in 12 of 20 communities (Figure 2). The highest number of detections (five of 34) occurred in Ontario (Malheur County); the largest proportion of detections (three of 14) was in Newport (Lincoln County). Eight of 12 communities with avian influenza A(H5) subtype detections are within counties that previously had an outbreak of HPAI in poultry. Only four of 12 communities with avian influenza A(H5) subtype detections had a licensed Grade A dairy processing facility or dairy farm within the sewershed. The avian influenza A(H5) subtype was not detected in wastewater from the two sewersheds with the highest number of licensed Grade A dairy processing facilities (Portland [seven]) and Grade A dairy farms (Tillamook [13]). No association between avian influenza A(H5) subtype detections in wastewater and location within a county with a history of poultry outbreak (p = 0.65) or location of licensed grade A dairy processing facilities or dairy farms within the sewershed (p = 0.65) was identified.

### Longitudinal Characterization

The avian influenza A(H5) subtype was detected during multiple weeks of each of the three influenza seasons during the study as well as during the summer of 2024 (Figure 1). The avian influenza A(H5) subtype was first detected in Oregon wastewater on March 21, 2022, approximately 7 weeks before avian influenza A(H5) was detected in wild birds (Figure 1) and approximately 6 weeks before HPAI was detected in poultry in Oregon (Figure 1).

## Discussion

Wastewater surveillance in Oregon first detected the avian influenza A(H5) subtype on March 21, 2022, 6 weeks before HPAI A(H5) was identified in an Oregon domestic poultry outbreak, 7 weeks before avian influenza A(H5) was identified through Oregon wild bird surveillance, and 2 years before HPAI A(H5N1) was detected in dairy cattle in the United States (*1*). In this retrospective analysis, avian influenza A(H5) subtype detections in wastewater were not associated with poultry outbreaks or the presence of licensed dairy processing facilities or farms within the sewershed. Importantly, many avian influenza A(H5) detections occurred before the spillover of the virus into dairy cattle, estimated to have occurred during November 2023–January 2024,^¶^ and no HPAI A(H5N1) outbreaks in dairy cattle have been identified in Oregon (*4*). These results do not support poultry or licensed dairy farm or processing facilities as the etiology of the avian influenza A(H5) subtype in Oregon wastewater and suggest that noncattle animals, suspected to be wild birds, are a significant animal contributor to wastewater within the state.

Oregon is located along the Pacific Flyway, a major north-south route for migratory birds in the Americas that extends from Alaska to Patagonia. An estimated 1 billion birds traverse the Pacific Flyway yearly, and Oregon contains many important stopover sites (*7*). Animal input can enter wastewater via stormwater in combined (i.e., open) sewersheds or via leaking pipes within separate (i.e., closed) sewersheds, as well as through the dumping of animal products into the sewer system (*6*,*8*). Surveillance programs that sample from wastewater clarifiers might also capture excreta from wild birds that have been observed using clarifiers and lagoons as resting habitats. The two communities in Oregon with the most avian influenza A(H5) subtype detections contain important habitats for migratory wild birds, including seasonal wetlands (Ontario) and estuaries of major rivers (Newport) (*7*,*9*). Avian influenza A(H5) subtype detections occurred in both combined and separate sewersheds.

### Limitations

The findings in this report are subject to at least two limitations. First, avian influenza A(H5) testing was performed retrospectively on samples that had tested positive for influenza A virus as part of Oregon’s routine influenza wastewater surveillance, which, before October 2023, occurred only during the influenza season. This approach limits the ability to describe seasonal avian influenza A(H5) subtype wastewater trends. Second, the testing methods used do not distinguish between animal sources or high- and low-pathogenic avian influenza A(H5) viruses (*10*).

### Implications for Public Health Practice

The timing and spatial clustering of avian influenza A(H5) subtype detections in Oregon wastewater suggest that noncattle animals, suspected to be wild birds, are important contributors of the virus to Oregon’s wastewater. Oregon’s first avian influenza A(H5) subtype detections in wastewater did not occur until after the introduction of the 2.3.4.4b clade into wild birds in North America and preceded Oregon’s first HPAI poultry outbreaks and avian influenza A(H5) detections through wild bird surveillance by more than 6 weeks. The first avian influenza A(H5) subtype detection in Oregon wastewater occurred almost 2 years before the multistate outbreak of HPAI A(H5N1) in dairy cattle, excluding cattle as a potential source for subtype detections before November 2023. On the basis of the results of this retrospective study, continued intermittent detections of the avian influenza A(H5) subtype in wastewater are anticipated, even in the absence of outbreaks in dairy cattle or occurrence of human cases. Wild birds, in which HPAI A(H5) is now enzootic, are an important consideration when interpreting avian influenza A(H5) subtype detections in wastewater. Wastewater surveillance, with consideration of all animal contributors and in conjunction with other surveillance metrics, has the potential to strengthen ongoing avian influenza surveillance efforts. 

## Footnotes

* Presence defined as a facility that produces Grade A milk under sufficiently sanitary conditions to qualify for human consumption. Grade A milk requires pasteurization and may be sold in all 50 states.

† A no-template control is a PCR reaction that does not contain a DNA or RNA template. This control checks for false positives that might occur because of cross-contamination.

§ 45 C.F.R. part 46.102(l)(2), 21 C.F.R. part 56; 42 U.S.C. Sect. 241(d); 5 U.S.C. Sect. 552a; 44 U.S.C. Sect. 3501 et seq.

¶
https://virological.org/t/preliminary-report-on-genomic-epidemiology-of-the-2024-h5n1-influenza-a-virus-outbreak-in-u-s-cattle-part-1-of-2/970
